# Genetic Diversity and Symbiotic Efficiency of Nodulating Rhizobia Isolated from Root Nodules of Faba Bean in One Field

**DOI:** 10.1371/journal.pone.0167804

**Published:** 2016-12-09

**Authors:** Lan Zou, Yuan Xue Chen, Petri Penttinen, Qin Lan, Ke Wang, Ming Liu, Dan Peng, Xiaoping Zhang, Qiang Chen, Ke Zhao, Xiangzhong Zeng, Kai Wei Xu

**Affiliations:** 1 Department of Microbiology, College of Resources, Sichuan Agricultural University, Chengdu, China; 2 Department of Environmental Sciences, University of Helsinki, Helsinki, Finland; 3 Soil and Fertilizer Institute, Sichuan Academy of Agricultural Sciences, Chengdu, China; Institute of Genetics and Developmental Biology Chinese Academy of Sciences, CHINA

## Abstract

Thirty-one nodulating rhizobium strains were collected from root nodules of spring and winter type faba bean cultivars grown in micro ecoarea, i.e. the same field in Chengdu plain, China. The symbiotic efficiency and phylogeny of these strains were studied. Effectively nitrogen fixing strains were isolated from both winter type and spring type cultivars. Based on phylogenetic analysis of 16S rRNA gene and concatenated sequence of *atpD*, *glnII* and *recA* genes, the isolates were assigned as *Rhizobium anhuiense* and a potential new *Rhizobium* species. The isolates were diverse on symbiosis related gene level, carrying five, four and three variants of *nifH*, *nodC* and *nodD*, respectively. Strains carrying similar gene combinations were trapped by both winter and spring cultivars, disagreeing with the specificity of symbiotic genotypes to reported earlier faba bean ecotypes.

## Introduction

Biological nitrogen fixation (BNF) is important for agriculture worldwide. Legume-rhizobia symbiosis accounts 60% of the total BNF [1, 2]. In the symbiosis, rhizobia form nodules on the roots or stems of the host plant. Rhizobia reduce atmospheric dinitrogen into ammonia inside the nodules.

Faba bean is grown worldwide as a source of protein and starch [3]. In 2013, the biggest faba bean producing countries were China, Australia, France and Egypt [4]. Besides yielding food and fodder, faba bean provides assimilated nitrogen to other crops grown in crop rotation [5]. Faba bean are divided into three main ecotypes: winter faba bean that is sown in autumn and grown in Mediterranean and the south of China, spring faba bean that is sown in spring and grown mostly in Europe and the north of China, and the Chinese Yunnan ecotype that is sown in both autumn and spring [6, 7]. In symbiosis, faba bean is considered as a selective legume. Previous reports indicated that the isolations from faba bean root nodules contain *Rhizobium leguminosarum* symbiovar *viciae* and *trifolii*, *R*. *etli*, *R*. *fabae*, *R*. *laguerreae*, *R*. *mesosinicum*, *R*. *anhuiense* and *Agrobacterium tumefaciens* [8–14]. The symbiotic genotype of the rhizobia determines the success in nodulating faba bean [15]. The faba bean ecotypes have been proposed to determine the distribution of rhizobial genomic and *nodC-nodD* types [9]. In addition, based on the phylogenetic analysis of *nodD* gene, faba bean symbionts were divided into groups roughly related to the division of winter and spring faba bean ecotypes [2].

In addition to the host cultivars, environmental conditions may affect the diversity of nodulating strains. Earlier we assessed the diversity of faba bean nodulating rhizobia on 21 sites with different environmental conditions across the Sichuan hilly areas, China [11]. For an insight into local diversity under uniform environmental conditions, and faba bean cultivars introduction experiment was coincidentally performed on one field in Sichuan agricultural university farm in Chengdu, so we collected nodules from fifteen faba bean cultivars grown on the same field in Chengdu, China, isolated nodulating rhizobia, and assessed their N_2_-fixation ability and phylogeny.

## Materials and Methods

### Isolation of the strains

Nodules were collected from fifteen faba bean cultivars grown on the research field of Sichuan Agricultural University in Chongzhou, Chengdu, Sichuan in 2013. Pink nodules were selected for isolation. The soil in the field was paddy soil with pH 6.3 and contained organic matter 37.6 g kg^-1^, total N 2.0 g kg^-1^, available N 136.0 mg kg^-1^, Olsen-P 20.4 mg kg^-1^, and exchangeable K 101.0 mg kg^-1^. After surface sterilization as described by Xu *et al*. [16], nodules were crushed and inoculated on YMA (yeast mannitol agar) medium supplemented with 25 mg L^-1^ congo red as described [17]. Isolates were purified by streaking on YMA medium and incubated at 28°C. Purified strains were maintained on YMA at 4°C for temporary storage and in 25% glycerol at -80°C for long-term storage.

### Plant nodulation and symbiotic efficiency test

The plant nodulation and symbiotic efficiency test was carried out using hydroponically grown native winter type cultivar Hanyuan Dabaidou. The seeds were surface sterilized with 95% ethanol (5 min) and 0.2% HgCl_2_ (3 min) followed by washing seven times with sterilized distilled water (5 min per time). Seeds were soaked overnight in sterilized water to soften the thick and hard seed coat. Sprouting, transplanting and inoculating were done as described previously by Xu *et al*. [16]. After 50 days, the plants were harvested and shoot dry mass and nodule numbers were measured. Excel 2010 (Microsoft, Redmond, USA) and SPSS 17.0 (SPSS Inc., Chicago, USA) were used to calculate the one-way analysis of variance with a least significant difference (LSD) analysis (*P* ≤ 0.05) of the mean values.

### Bacterial DNA extraction and PCR-RFLP of 16S rRNA gene and intergenic spacer region (IGS)

DNA was extracted as described by Little [18]. Primer pair P1 and P6 was used for amplification of 16S rRNA gene [11]. IGS was amplified using primer pair pHr (F) and p23SR01(R) [11]. Target sequences were amplified in Bio-RAD MyCycler^TM^ with 20 pmol of each primer pair and 50 ng total DNA as template using a Golden Easy PCR System (TIANGEN, Beijing, China). The PCR protocols were as described by Xu *et al*. [16], except that the annealing temperature was 60°C for IGS.

16S rRNA gene and IGS PCR-RFLP were done using restriction endonucleases *Taq*I, *Hae*III, *Hin*fI and *Msp*I. 5 μl of PCR product was digested by the restriction endonucleases separately in a 10 μl reaction volume following the manufacturer’s instructions (Fermentas, EU). The fragments were separated in 2% agarose gel containing 0.5 μg ml^-1^ ethidium bromide at 80 V for 3 hours in 1×TAE (Tris-Acetate-EDTA) buffer. Gels were documented with a Gel Document System (Bio-rad, USA). 16S RFLP and IGS RFLP analyses were done using the UPGMA clustering algorithm of NTSYSpc program [19].

### Sequence analyses

Eight isolates were selected for sequencing of housekeeping and symbiosis related genes. 16S rRNA gene, *atpD*, *glnII*, *recA*, *nifH*, *nodC* and *nodD* were amplified using the primer pairs P1/P6, atpDF3/atpDR [20], glnII5/glnII6 [21], recAF3/recAR3 [20], nifHctg/nifHR [22], nodCF/nodCR [23] and NBA12F/NBA12R [24], respectively, using the related protocols. The PCR products were directly sequenced at BGI Tech (Shenzhen, China). The sequences were compared with sequences in the NCBI database using BlastN to find reference sequences. The reference sequences and the sequences from the representative strains were aligned using ClustalW in MEGA 6.0 [25]. Phylogenetic trees were built using Neighbor-joining (NJ) method with 1000 resampling bootstrapping in MEGA 6.0. The percentage similarity of the genes was estimated using Distance in MEGA 6.0 [16]. In the multilocus sequence analysis of the concatenated housekeeping genes *atpD*, *recA* and *glnII*, we applied 97% similarity as the threshold for defining genospecies [26, 27].

## Results

### Isolation and symbiotic efficiency

Rhizobial bacteria were isolated from four spring type and eleven winter type faba bean cultivars grown in a single field in Chengdu plain (Table 1). The isolates formed nodules on the root of the native faba bean Hanyuan Dabaidou with the average nodule numbers ranging from 19 to 110 per plant. Compared to the uninoculated control, nine isolates increased significantly (*P* ≤ 0.05) the shoot dry mass of the plant, and were considered as potential inoculant strains (Table 1). The potential inoculant strains were isolated from three local Sichuan winter type faba bean cultivars and three spring type faba bean cultivars.

**Table 1:** Rhizobial strains isolated in this study and their symbiotic and genotypic characteristics.

Strain^a^	Host cultivar^b^	Cultivar origin	Cultivar ecotype	Shoot dry mass	No. of nodules	16S-RFLP genotype^d^	IGS-RFLP genotype^e^
				(g plant^-1^)^c^	per plant		
SCAUf68	DB	Sichuan	Winter	0.924±0.0724	53	A	XI
SCAUf65	HN	Henan	Spring	1.049±0.0621↑^*^	36.7	A	XI
SCAUf64	HN	Henan	Spring	0.696±0.0214	62	A	VII
SCAUf73	JX	Shanxi	Spring	1.176±0.0922↑^*^	52.7	A	X
SCAUf76	JX	Shanxi	Spring	1.019±0.0643↑^*^	64.3	A	XII
SCAUf75	JX	Shanxi	Spring	0.849±0.144	40.7	A	XII
SCAUf74	JX	Shanxi	Spring	0.844±0.0767	22	A	XI
SCAUf70	LP	Shandong	Spring	1.073±0.0860↑^*^	19.3	A	XI
SCAUf72	LP	Shandong	Spring	0.962±0.0656	54.7	A	XI
SCAUf71	LP	Shandong	Spring	0.685±0.0447	98.3	A	IX
SCAUf55	S1	Sichuan	Winter	0.829±0.264	87	A	II
SCAUf59	S2	Sichuan	Winter	1.006±0.0872	50.3	A	XI
SCAUf57	S2	Sichuan	Winter	0.907±0.0809	47.7	A	XI
SCAUf58	S2	Sichuan	Winter	0.899±0.138	44	A	XI
SCAUf56	S2	Sichuan	Winter	0.650±0.0893	38.3	A	XI
SCAUf61	S3	Sichuan	Winter	1.201±0.0914↑^*^	21.3	A	XII
SCAUf60	S3	Sichuan	Winter	1.035±0.168↑^*^	69.3	A	XII
SCAUf62	S3	Sichuan	Winter	0.862±0.141	59	A	VI
SCAUf63	S4	Sichuan	Winter	0.718±0.245	74.3	A	XII
SCAUf77	S5	Sichuan	Winter	1.051±0.255↑^*^	111.3	A	XII
SCAUf78	S5	Sichuan	Winter	0.899±0.0552	46	A	I
SCAUf79	S5	Sichuan	Winter	0.764±0.210	45	A	XII
SCAUf81	S6	Sichuan	Winter	1.054±0.190↑^*^	124.3	A	IV
SCAUf80	S6	Sichuan	Winter	1.013±0.0880↑^*^	114	A	IV
SCAUf82	S7	Sichuan	Winter	0.819±0.0187	19.7	A	VIII
SCAUf83	S8	Sichuan	Winter	0.787±0.0987	84.3	A	XI
SCAUf84	S9	Sichuan	Winter	0.845±0.158	14	A	V
SCAUf85	S9	Sichuan	Winter	0.636±0.0633	17.7	A	XIII
SCAUf67	YCDB	Zhejiang	Winter	0.925±0.0691	40.5	A	XI
SCAUf66	YCDB	Zhejiang	Winter	0.883±0.0235	34	A	XI
SCAUf69	ZZL	Tianjin	Spring	0.980±0.0664	110.3	A	III
NC	-	-	-	0.661±0.115	-	-	-

^a^ The strains were deposited in Sichuan Agricultural University (SCAU) strain collection center. NC = uninoculated control in the symbiotic efficiency test.

^b^ Faba bean cultivars: S1-S9 = Local Sichuan faba bean cultivars; JX = Jingxuan; LP = Lüpi; HN = Henan; ZZL = Zhenzhulü; YCDB = Yicundabai; DB = Dabai.

^c^↑^*^ = the plant dry weight was significantly higher than that of NC according to the LSD test (P = 0.05). Data is shown as average ± standard error (n = 3).

^d,e^ 16S-RFLP and IGS-RFLP genotype represents the combination of restriction patterns obtained by enzymes used (*Hae*III, *Hin*fI, *Msp*I, and *Taq*I).

### RFLP analyses

**Fig 1:**
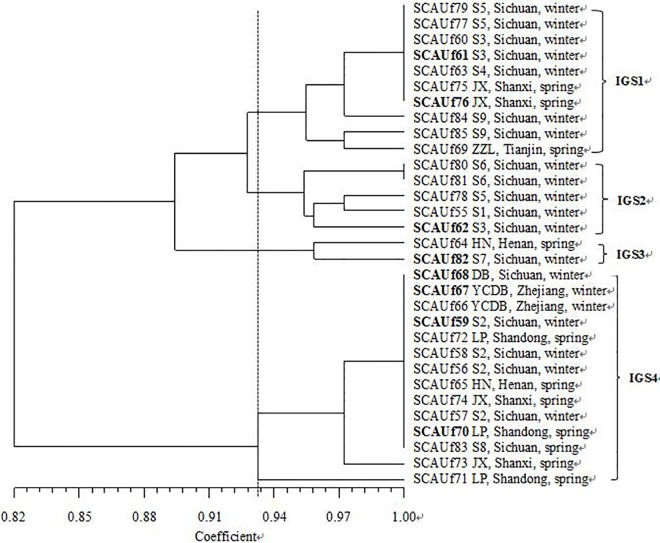
IGS PCR-RFLP analysis by four restriction endonucleases (*Msp*I, *Hin*fI, *Hae*III and *Taq*I) of strains isolated from faba bean nodules. Strain number is followed by the name, the origin and the ecotype of the host of isolation. Abbreviations are as in the footnote of Table 1. The IGS PCR-RFLP groups are labeled with Roman numerals I to III. Isolates in **bold** were selected for sequencing of housekeeping and symbiosis genes.

The 16S rRNA gene of all the 31 isolates was successfully amplified and approximately 1500 bp single band was obtained. In the 16S rRNA gene RFLP analysis all the strains represented the same 16S genotype (Table 1). In the IGS PCR, one band was amplified from all the isolates except SCAUf57, SCAUf59, SCAUf66 and SCAUf69 for which two bands were detected. According to the IGS-RFLP, the isolates represented thirteen genotypes that were divided into four IGS groups with 10, 5, 2, and 14 isolates at 93% similarity (Fig 1). The nine significantly growth promoting isolates were distributed into groups IGS1 (4 strains), IGS2 (2 trains) and IGS4 (3 strains).

### Phylogenetic analysis of 16S rRNA gene

**Fig 2:**
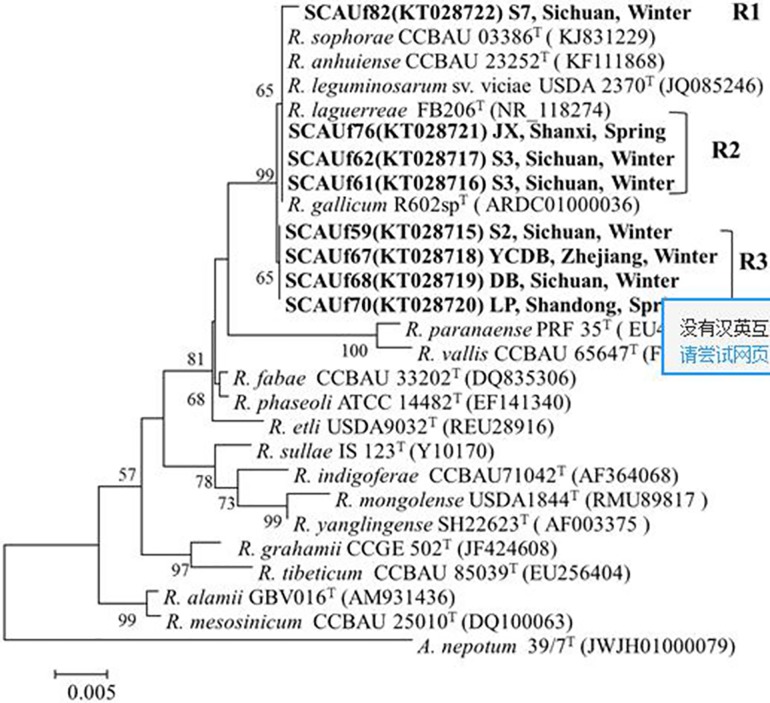
The phylogenetic relationships between the representative faba bean strains and reference strains based on 16S rRNA gene sequences (1278 nt). Genbank accession numbers are in parentheses. Bootstrap values above 50% are shown on the branches. Scale bar represents 0.2% nucleotide substitutions. *R*: *Rhizobium*.

Based mainly on IGS RFLP while also considering the hosts of isolation, eight representative strains were selected for sequencing of the housekeeping and symbiosis genes. In accordance with the IGS RFLP (Fig 1), the strains were divided into three groups related to *Rhizobium* in the 16S rRNA gene phylogenetic tree (Fig 2). SCAUf82 clustered with *R*. *leguminosarum* USDA 2370^T^ with 99.8% similarity (group R1). SCAUf61, SCAUf62 and SCAUf76 clustered with *R*. *laguerreae* FB206^T^, *R*. *leguminosarum* USDA 2370^T^, *R*. *anhuiense* CCBAU 23252^T^, *R*. *sophorae* CCBAU 03386^T^ and *R*. *gallicum* R602sp^T^ with 100% similarity (group R2). SCAUf59, SCAUf67, SCAUf68 and SCAUf70 clustered separately (group R3).

### Phylogenetic analyses of housekeeping genes

**Fig 3:**
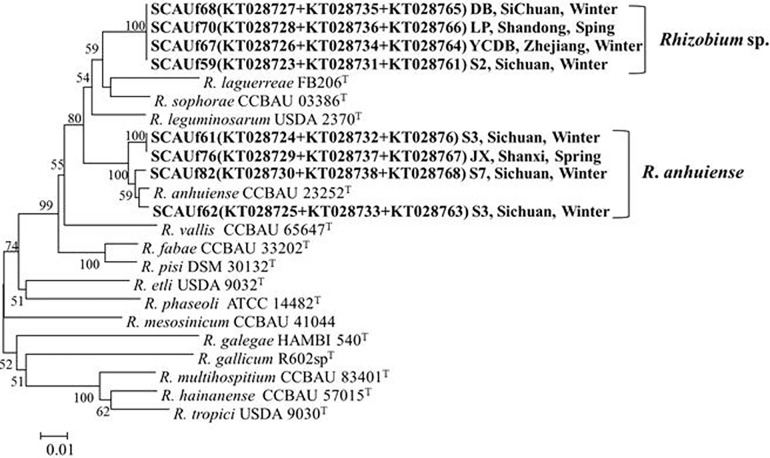
The phylogenetic relationships between the representative faba bean strains and reference strains based on multilocus sequence analysis (MLSA) of *atpD* (411 nt), *glnII* (514 nt), and *recA* (384 nt) genes. Genbank accession numbers are in parentheses. Bootstrap values above 50% are shown on the branches. Scale bar represents 1% nucleotide substitutions. *R*: *Rhizobium*.

Three housekeeping genes (*atpD*, *glnII* and *recA*) were successfully amplified from all representative strains. In the multilocus sequence analysis (MLSA) tree that was based on concatenated gene sequences (Fig 3) and single gene trees (S1–S3 Figs) the representative strains were divided into two clusters related to *Rhizobium*. SCAUf59, SCAUf67, SCAUf68 and SCAUf70 were 96.6% similar with the type strain *R*. *sophorae* CCBAU 03386^T^ and were assigned as *Rhizobium* sp. SCAUf61, SCAUf62, SCAUf76, and SCAUf82 clustered with *R*. *anhuiense* CCBAU 23253^T^ with 98.8% to 99.3% similarity in the MLSA, and were thus assigned as *R*. *anhuiense*.

### Phylogenetic analyses of symbiosis genes

**Fig 4:**
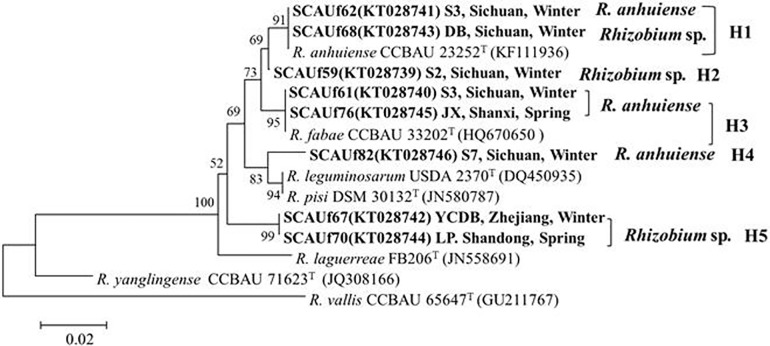
Phylogenetic tree based on *nifH* (346 nt) gene of representative faba bean strains and reference strains. Genbank accession numbers are in parentheses. Bootstrap values above 50% are shown on the branches. Scale bar represents 1% nucleotide substitutions. *R*: *Rhizobium*.

Approximately 700 bp, 900 bp and 1500 bp fragments of *nifH*, *nodC* and *nodD*, respectively, were amplified from all the representative strains. In the *nifH* phylogenetic tree, the strains were distributed into five groups (Fig 4). The *Rhizobium* sp. SCAUf68, *R*. *anhuiense* SCAUf62 and *R*. *anhuiense* CCBAU 23252^T^ *nifH* genes in the group H1 were 100% similar. *Rhizobium* sp. strain SCAUf59 clustered alone as group H2. In the group H3, *R*. *anhuiense* strains SCAUf61 and SCAUf76 carried a *nifH* gene 100% similar with *R*. *fabae* CCBAU 33202^T^. *R*. *anhuiense* strain SCAUf82 clustered with *R*. *leguminosarum* USDA 2370^T^ and *R*. *pisi* DSM 30132^T^ with 98.4% similarity in group H4. *Rhizobium* sp. strains SCAUf67 and SCAUf70 clustered separately in group H5.

**Fig 5:**
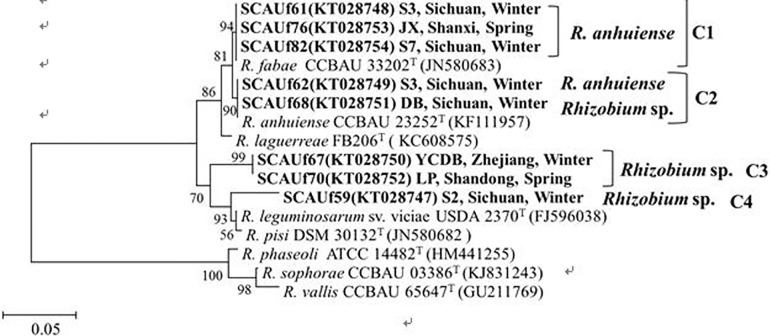
Phylogenetic tree based on *nodC* (534 nt) gene of representative faba bean strains and reference strains. Genbank accession numbers are in parentheses. Bootstrap values above 50% are shown on the branches. Scale bar represents 2% nucleotide substitutions. *R*: *Rhizobium*.

In the *nodC* phylogenetic tree, the strains were distributed into four groups (Fig 5). *R*. *anhuiense* strains SCAUf61, SCAUf76 and SCAUf82 clustered with *R*. *fabae* CCBAU 33202^T^ in group C1. *R*. *anhuiense* SAUf62 and *Rhizobium* sp. SCAUf68 clustered with *R*. *anhuiense* CCBAU 23252^T^ with 100% similarity in group C2. *Rhizobium* sp. strains SCAUf67 and SCAUf70 clustered separately as group C3. SCAUf59 clustered with *Pisum sativum* symbionts in group C4 (Fig 5).

**Fig 6:**
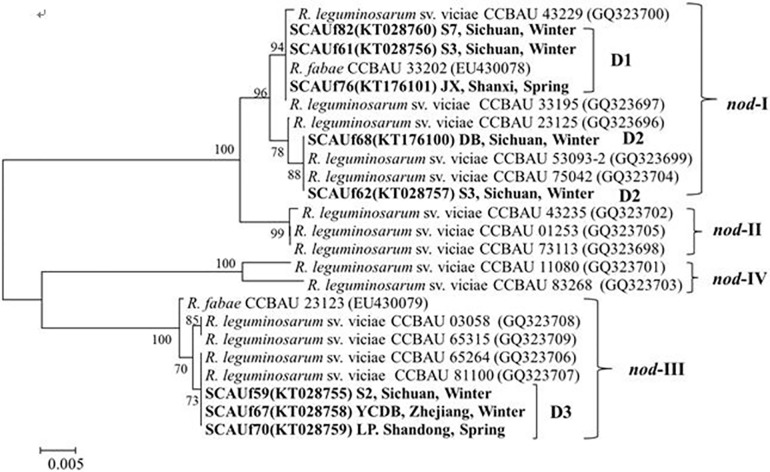
Phylogenetic tree based on *nodD* (847 nt) gene of representative faba bean strains and reference strains. Genbank accession numbers are in parentheses. Bootstrap values above 50% are shown on the branches. Scale bar represents 0.5% nucleotide substitutions. *R*: *Rhizobium*.

The *nodD* sequences were divided into three groups (Fig 6). *R*. *anhuiense* strains SCAUf61, SCAUf76 and SCAUf82 clustered with *R*. *fabae* CCBAU 33202^T^ with 100% similarity in group D1. *R*. *anhuiense* SCAUf62 and *Rhizobium* sp. SCAUf68 carried *nodD* genes 100% similar to that of CCBAU 53093–2 in group D2. *Rhizobium* sp. strains SCAUf59, SCAUf67 and SCAU70 clustered with *R*. *leguminosarum* CCBAU 65264 and CCBAU 81100 with 100% similarity in group D3 (Fig 6).

## Discussion

Biological N_2_ fixation (BNF) is a key component in sustainable agriculture since BNF can replace nitrogen fertilizer in growing legume crops. Improving legume yields requires efficiently nitrogen-fixing symbiotic bacteria, rhizobia. Efficient rhizobium strains may be selected and applied as inoculants to increase yields [20, 28].

Faba bean cultivars are divided into three ecotypes: winter faba bean, spring faba bean and Yunnan ecotype [6, 7]. Based on the sequence analysis of symbiosis gene *nodD*, faba bean symbionts were divided into groups roughly related to the division of their hosts to winter and spring ecotypes [2]. The faba beans grown in Sichuan are the winter type. Earlier we isolated faba bean rhizobia from 21 sites covering most of the faba bean growing area in Sichuan [11]. The nodulating strains carried *nodC* similar to those of strains that nodulate winter type faba bean and field pea [11]. In the present study, we collected 31 rhizobium strains from root nodules of fifteen faba bean cultivars grown on the same field. The growth of a local faba bean cultivar was significantly promoted by nine isolates, implying that these strains were potential inoculants. Potential inoculant strains were isolated from both winter type and spring type cultivars. The applicability of these strains must be further tested in field experiments.

Faba beans are mainly nodulated by *R*. *leguminosarum*, *R*. *etli*, *R*. *fabae* and *R*. *anhuiense* [9, 10, 13, 29]. In our earlier study we found that in Sichuan, in addition to *R*. *leguminosarum*, faba bean was nodulated by five *Rhizobium* species [11]. *R*. *leguminosarum* strains are quite diverse, and the Sichuan *R*. *leguminosarum* strains were divided to two distantly related groups [11, 30]. In this study, based on both 16S rRNA gene and multilocus sequence analyses of three housekeeping genes, the representative isolates were assigned as *R*. *anhuiense* and a potential new *Rhizobium* species.

16S rRNA gene and housekeeping gene sequence analyses are usually applied to assess the phylogenetic position of rhizobia, whereas analyses of symbiosis related genes reveal the symbiotic genotype of rhizobia. Comparison of housekeeping and symbiosis genes gives crucial information on how rhizobia evolve [31]. The symbiosis related genes are commonly on mobile genetic elements that are transferrable between strains [32]. The *nifH* gene, encoding the nitrogenase Fe protein, is essential for symbiotic nitrogen fixation [33]. The *nodC* is essential for the synthesis of the aminosugar backbone of rhizobial signaling molecules [34].The *nodD* encodes a transcriptional regulator of *nod* genes [2]. The faba bean nodulating Sichuan *R*. *leguminosarum* strains carried three different *nifH* and *nodC* variants [11]. In addition to them, we found two and one new variants of *nifH* and *nodC*, respectively. Legumes may be classified as promiscuous or selective according to the number of rhizobia they are compatible with. For example, in Sichuan the promiscuous *Leucaena leucocephala* was nodulated by seven rhizobial species [35]. However, on nodulation gene (*nodC*) level there was less diversity [16]. Interestingly, the species and nodulation gene diversity of the faba bean symbionts was the opposite: two species that carry four variants of *nodC*, raising an intriguing question on what ultimately governs the host specificity. Both *R*. *anhuiense* and *Rhizobium* sp. included strains with respective three different *nifH*-*nodC* combinations (Table 2), indicating that the genes had been horizontally transferred (Table 2). The *nodD* variants D3 and D1were found together with two different *nifH*-*nodC* combinations suggesting a possible partial transfer of symbiotic island or plasmid (Table 2).

**Table 2:** The phylogenetic relationships of strains isolated from fifteen faba bean cultivars grown on the same field in Chengdu, China.

Strain^a^	IGS group	The most similar species (sequence similarity with type strain, %)^b^					
		*atpD*	*glnII*	*recA*	MLSA	*nodC*	*nifH*
***R*. *anhuiense*** (17)							
**SCAUf61**, 60, 63, 69, 75,	IGS1	Ra(98.5%)	Ra(99.6%)	Ra(98.2%)	Ra(98.8%)	Rf(100%)	Rf(100%)
**SCAUf76,** 77, 79, 84, 85		Ra(98.5%)	Ra(99.6%)	Ra(98.2%)	Ra(98.8%)	Rf(100%)	Rf(100%)
**SCAUf62**, 55, 78, 80, 81	IGS2	Ra(99.7%)	Ra(99.2%)	Ra(98.8%)	Ra(99.3%)	Ra(100%)	Ra(100%)
S**CAUf82**, 64	IGS3	Ra(99.7%)	Ra(98.3%)	Ra(100%)	Ra(99.3%)	Rf(100%)	Rle(98.4%)
***Rhizobium* sp.**(14)							
**SCAUf59**, 56, 57, 58	IGS4	Rla(95.5%)	Rle(98.1%)	Rle(97.6%)	Rs(96.6%)	Rle(96.0%)	Ra(99.4%)
**SCAUf67**, 65, 66		Rla(95.5%)	Rle(98.1%)	Rle(97.6%)	Rs(96.6%)	Rle(95.3%)	Rle(96.8%)
**SCAUf68**, 74, 83		Rla(95.5%)	Rle(98.1%)	Rle(97.6%)	Rs(96.6%)	Ra(100%)	Ra(100%)
**SCAUf70**, 71, 72, 73		Rla(95.5%)	Rle(98.1%)	Rle(97.6%)	Rs(96.6%)	Rle(95.3%)	Rle(96.8%)

^a^ Strains in this study(number of isolates). The isolates in bold face were representative strains and were used in 16S rRNA, *recA*, *glnII*, *atpD*, *nifH*, and *nodC* genes sequencing.

^b^ MLSA: Multilocus sequence analysis of concatenated *atpD*, *glnII* and *recA* genes. Ra = *R*. *anhuiense*; Rf = *R*. *fabae*; Rla = *R*. *laguerreae*; Rle = *R*. *leguminosarum*; Rs = *R*. *sophorae*.

The genotypes *nod-*I and *nod-*III are the dominant *nodD* genotypes of winter type and Yunnan-China type faba beans, respectively [2, 29]. In our study, the isolates carried three *nodD* variants. The variants D1 and D2 clustered with *nod-*I, and the variant D3 with *nod-*III. Strains carrying similar *nodD* gene variants and *nifH*-*nodC* combinations were trapped by both winter and spring cultivars, disagreeing with the division of *nodD* to cultivar specific variants. Obviously, analysis of a greater number of strains is needed to conclude whether the different cultivars favor certain *nodD* gene variants. Instead of cultivar specificity, the division of *nodD* types may be geographical. Since Sichuan is in between the spring type, winter type and Yunnan type faba bean cultivation areas, finding rhizobia carrying different *nodD* genotypes may be expected.

In all, the soil hosted *R*. *anhuiense* and a potential new *Rhizobium* species that were diverse on the symbiosis related gene level. The strains nodulated both winter and spring type cultivars. Even though the rhizobia may be co-introduced with the host [36], isolating similar strains from both winter and spring type cultivars indicates that the strains originated from soil, not seed. The local genetic diversity under uniform environmental conditions was equal to diversity of nodulating strains collected from many sampling sites with different conditions and cultivars, indicating that the symbiotic specificity of the faba bean rhizobia was related to the cultivars.

## Supporting Information

S1 Fig Phylogenetic tree based on *atpD* (396 nt) genes of the representative strains isolated from fababean and reference strains. Genbank accession numbers are in parentheses. Bootstrap values ≥ 50% areshown on the branches. Scale bar represents 1% nucleotide substitutions. *R*: *Rhizobium*.
(JPG)

S2 Fig Phylogenetic tree based on *glnII* (483 nt) genes of the representative strains isolated from fababean and reference strains. Genbank accession numbers are in parentheses. Scale bar represents 1%nucleotide substitutions. Bootstrap values ≥ 50% are shown on the branches, *R*: *Rhizobium*.
(JPG)

S3 Fig Phylogenetic tree based on *recA* (345 nt) genes of the representative strains isolated from fababean and reference strains. Genbank accession numbers are in parentheses. Bootstrap values ≥ 50% areshown on the branches. Scale bar represents 1% nucleotide substitutions. *R*: *Rhizobium*.
(JPG)
