# Supplementary figures and images for: Genetic Diversity and Symbiotic Efficiency of Nodulating Rhizobia Isolated from Root Nodules of Faba Bean in One Field
Source: PLoS One. 2016 Dec 9;11(12):e0167804. doi: 10.1371/journal.pone.0167804 (PMC5147995; doi:10.1371/journal.pone.0167804)

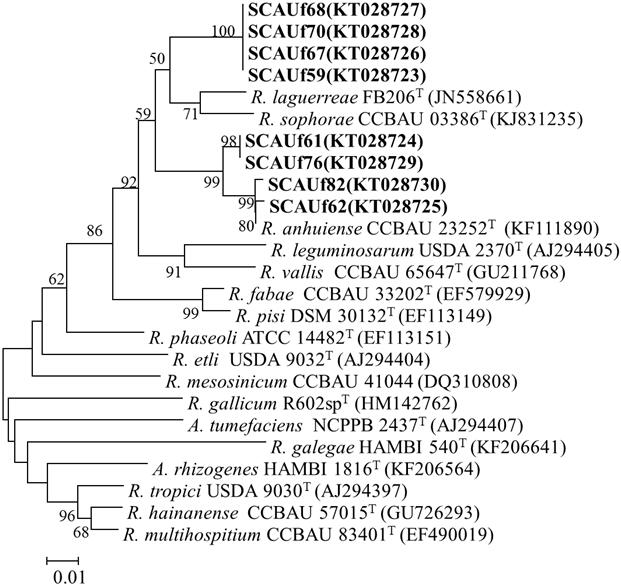

Supplement: S1 Fig — Genbank accession numbers are in parentheses. Bootstrap values ≥ 50% areshown on the branches. Scale bar represents 1% nucleotide substitutions. R: Rhizobium. (JPG) [file pone.0167804.s001.jpg]

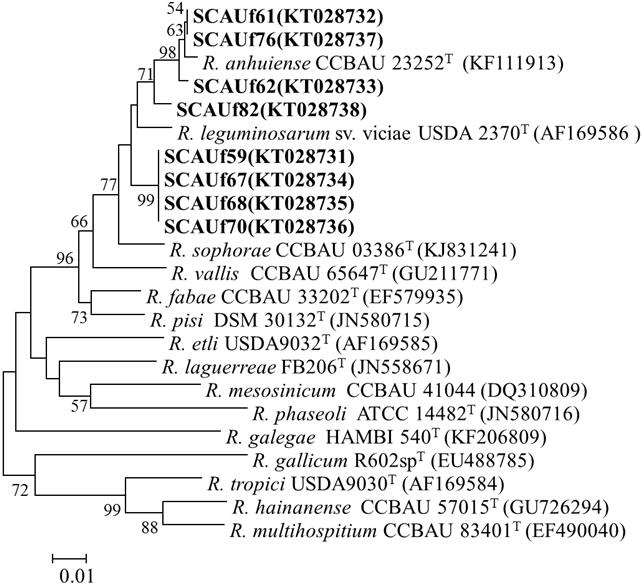

Supplement: S2 Fig — Genbank accession numbers are in parentheses. Scale bar represents 1%nucleotide substitutions. Bootstrap values ≥ 50% are shown on the branches, R: Rhizobium. (JPG) [file pone.0167804.s002.jpg]

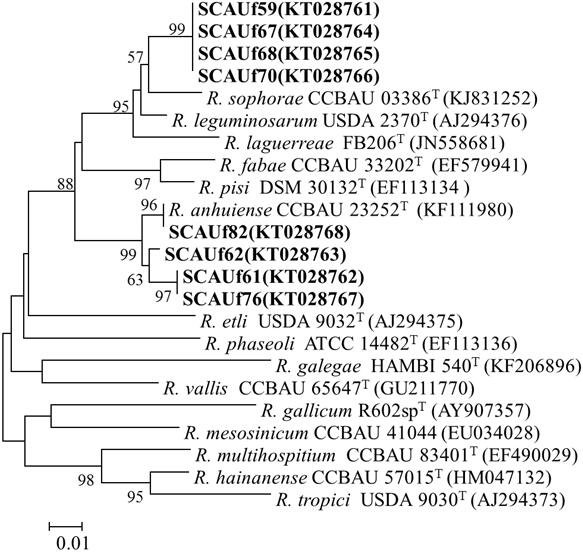

Supplement: S3 Fig — Genbank accession numbers are in parentheses. Bootstrap values ≥ 50% areshown on the branches. Scale bar represents 1% nucleotide substitutions. R: Rhizobium. (JPG) [file pone.0167804.s003.jpg]
